# Complete androgen insensitivity syndrome caused by a deep intronic pseudoexon-activating mutation in the androgen receptor gene

**DOI:** 10.1038/srep32819

**Published:** 2016-09-09

**Authors:** Johanna Känsäkoski, Jarmo Jääskeläinen, Tiina Jääskeläinen, Johanna Tommiska, Lilli Saarinen, Rainer Lehtonen, Sampsa Hautaniemi, Mikko J. Frilander, Jorma J. Palvimo, Jorma Toppari, Taneli Raivio

**Affiliations:** 1 Physiology, Faculty of Medicine, University of Helsinki, Helsinki, Finland; 2 Children’s Hospital, Helsinki University Hospital, Helsinki, Finland; 3 Department of Pediatrics, University of Eastern Finland and Kuopio University Hospital, Kuopio, Finland; 4 Institute of Dentistry and Institute of Biomedicine, University of Eastern Finland, Kuopio, Finland; 5 Research Programs Unit, Genome-Scale Biology, Faculty of Medicine, University of Helsinki, Helsinki, Finland; 6 Institute of Biotechnology, University of Helsinki, Helsinki, Finland; 7 Institute of Biomedicine, University of Eastern Finland, Kuopio, Finland; 8 Departments of Physiology and Pediatrics, University of Turku and Turku University Hospital, Finland

## Abstract

Mutations in the X-linked androgen receptor (*AR*) gene underlie complete androgen insensitivity syndrome (CAIS), the most common cause of 46,XY sex reversal. Molecular genetic diagnosis of CAIS, however, remains uncertain in patients who show normal coding region of *AR*. Here, we describe a novel mechanism of AR disruption leading to CAIS in two 46,XY sisters. We analyzed whole-genome sequencing data of the patients for pathogenic variants outside the *AR* coding region. Patient fibroblasts from the genital area were used for *AR* cDNA analysis and protein quantification. Analysis of the cDNA revealed aberrant splicing of the mRNA caused by a deep intronic mutation (c.2450-118A>G) in the intron 6 of *AR*. The mutation creates a de novo 5′ splice site and a putative exonic splicing enhancer motif, which leads to the preferential formation of two aberrantly spliced mRNAs (predicted to include a premature stop codon). Patient fibroblasts contained no detectable AR protein. Our results show that patients with CAIS and normal *AR* coding region need to be examined for deep intronic mutations that can lead to pseudoexon activation.

Androgen insensitivity syndrome (AIS) is the most common known cause of 46,XY disorders of sex development, ranging from mild (MAIS) and partial (PAIS) to complete (CAIS) forms of androgen resistance. CAIS is characterized by a female phenotype in a genetically male (46,XY) individual, whereas PAIS ranges from predominantly female to predominantly male phenotype, and MAIS patients have normal external male genitalia but they may suffer from infertility due to defective spermatogenesis^1^. Mutations of variable severity in *AR*, the X-linked gene encoding the androgen receptor, cause the different forms of AIS, which total more than 500 mutations reported by 2012 in the Androgen Receptor Gene Mutations Database^2^. About 80 to 100% of CAIS patients are found to have an *AR* mutation^3^, whereas in PAIS, the percentage may be as low as 16%^4^. The identification of a pathogenic mutation in *AR* confirms the diagnosis of AIS, especially in the milder forms which have some phenotypic overlap with other disorders of sex development^5^.

Androgen receptor mutations are mostly missense and enriched in the ligand-binding domain (LBD) of the protein^3^. Frameshift, nonsense, and splice-site mutations also cause the disorder in several patients. The fact that a mutation in *AR* has not even been identified in all patients with the complete form of the disorder raises the possibility of mutations in necessary AR cofactor(s) as the underlying cause in some cases^6^. Such mutations, however, are yet to be discovered^3^.

Here, we demonstrate a new mechanism for CAIS. We show that a deep intronic pseudoexon-activating mutation in the intron between exons 6 and 7 of *AR*, detected in two siblings with CAIS with normal *AR* coding region and conserved splice sites, leads to aberrant splicing of the *AR* mRNA and insufficient AR protein production.

## Results

### Sequencing and cDNA analysis of AR

**Figure 1:**
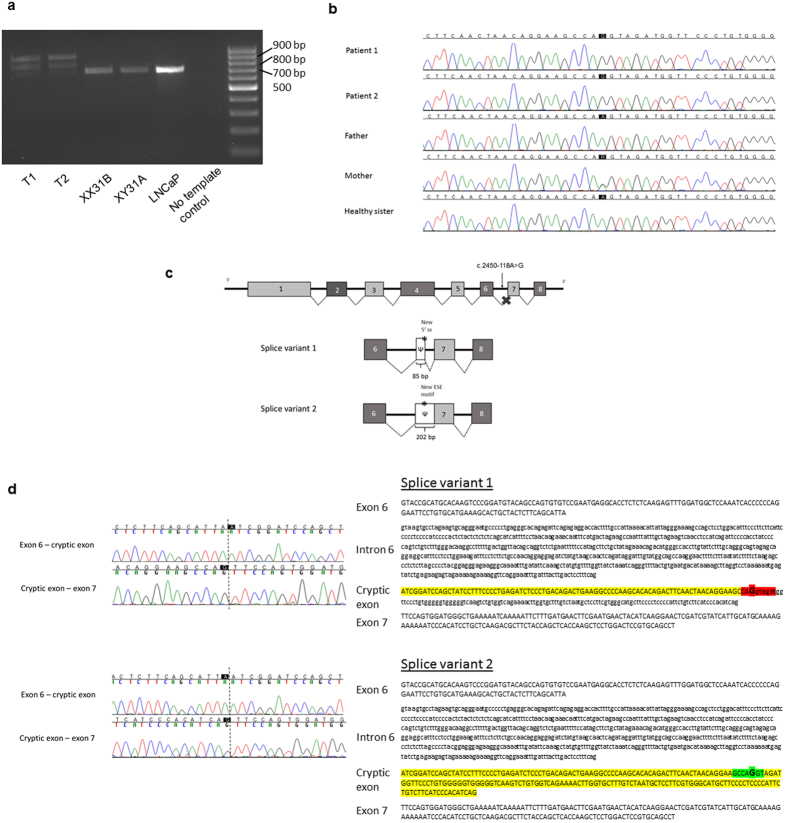
The PCR amplification of *AR* cDNA (**a**), the identified intronic *AR* mutation (**b**), the schematic representation of the two aberrant mRNAs caused by the deep intronic mutation (**c**), and the nucleotide sequences of exons 6 and 7 and intron 6 (**d**). (**a**) The *AR* cDNA was PCR-amplified with primers on exon 5 and the 3′ UTR and the products were visualized on a 1.5% agarose gel. Lanes T1 and T2 are amplification products of the patient samples, XX31B and XY31A are control fibroblast samples, and LNCaP is from prostatic cancer cells. (**b**) The sequence chromatograms showing the intronic mutation which was confirmed to be present as hemizygotic in both patients and heterozygotic in the mother, whereas it was absent in the father and the healthy sister. (**c**) Schematic drawing of the aberrant *AR* pre-mRNA splicing leading to the two longer mRNAs. The cryptic exons are marked with Ψ, and the mutation site is marked with an asterisk. (**d**) On the left are shown the sequence chromatograms showing the borders of the cryptic exonic sequences and the normal exons derived from sequencing of the gel-extracted aberrant PCR products. On the right are shown the nucleotide sequences of exon 6, intron 6, and exon 7, including the c.2450-118A>G mutation marked in bold and in bigger font size. For splice variant 1, the predicted donor site motif is highlighted in red. For splice variant 2, the predicted SRSF1 binding motif is highlighted in green. The intronic sequences that are spliced into the mRNA are in uppercase and highlighted in yellow (or red/green for sequences that are part of a splicing motif) and correspond to the boxes marked with Ψ in panel C. Normal exonic sequences are in uppercase and are not highlighted, intronic sequences are in lowercase.

Sanger-sequencing of the *AR* coding sequence from genomic DNA of the two siblings with CAIS revealed only one synonymous polymorphism [c.639G>A p.(Glu213=), rs6152, minor allele frequency 0.24 in the 1000 Genomes (www.1000genomes.org; phase 3, all variants) database]. No rare potentially pathogenic variants in the coding region or in the conserved splice sites of the gene were found. PCR-amplification of the *AR* cDNA from the patients’ genital fibroblasts with primers situated on exon 5 and the 3′UTR, however, revealed abnormally long products (approximately 800 and 900 bp) in comparison to controls (Fig. 1a). In addition, the normal-sized product of 683 bp was much fainter in the patient samples than in the controls (Fig. 1a). These findings suggested a splicing defect caused by a deep intronic mutation between exons 5 and 8.

Whole-genome sequencing produced sequencing data with an average sequencing depth of at least 35.14 and 99.68% coverage for each sample, and at least 19.04 average sequencing depth (at least 99.4% bases with ≥4x coverage) in the *AR* gene region (GRCh37 genomic position chrX:66,764,465–66,950,461) for each DNA sample. Consistent with the result from *AR* cDNA PCR-amplification, the sequencing data revealed a point mutation in intron 6 (GRCh37 genomic location chrX:66,942,551, c.2450-118A>G) that was not reported in variation databases [dbSNP (http://www.ncbi.nlm.nih.gov/projects/SNP/index.html) and 1000 Genomes]. This mutation was confirmed by Sanger-sequencing to be present as hemizygotic in both affected sisters and heterozygotic in the mother, whereas it was absent in the father and the healthy sister (Fig. 1b). No other potentially pathogenic mutations in the *AR* gene were found in the whole-genome sequencing data.

### *In silico* analysis

*In silico* analysis with Human Splicing Finder suggested that the mutation leads to the formation of potential splice sites (ss), either a cryptic acceptor site [+61.08% increase in the motif score from 47.4 (wt), to 76.35 (mut) with the HSF prediction algorithm (consensus value (CV) threshold 65, variation threshold +/−10%)] or a donor site [+15.34% increase of the score from 68.92(wt) to 79.49 (mut) with the HSF prediction algorithm (CV threshold 65, variation threshold +/−10%), and +1053.95% increase from 0.76 (wt) to 7.25 (mut) with MaxEntScan algorithm (CV threshold 3, variation threshold +/−30%)]. Additionally, the analysis suggested that the mutation may also create a new exonic splicing enhancer (ESE) motif specific for the SR-family protein SRSF1 (SF2/ASF) [mutant motif value 75.19 with ESE Finder algorithm (threshold value 72.98)].

### Sequencing of the AR cDNA

Sequencing of the two aberrantly spliced RT-PCR products (Fig. 1a) revealed 85 and 202 bp insertions corresponding to a cryptic exon inclusion between exons 6 and 7 (Fig. 1c). Both aberrant products share the same splice acceptor site 84 bp upstream of the mutation site (Fig. 1d). With the smaller product, a donor site (5′ splice site; 5′ss) is activated immediately downstream of the mutation site, which leads to an inclusion of an 85 bp cryptic exon between exons 6 and 7. With the long product, the 5′ss downstream of the mutation site is not used, which results in a longer exon 7 that contains upstream intronic sequences (Fig. 1d). Together, the RT-PCR analyses support the *in silico* predictions and indicate that the mutation leads to the formation of a novel 5′ss (shorter product) and an ESE (longer product), which both subsequently promote the use of the upstream 3′ss shared between the cryptic transcripts.

### AR protein analysis

**Figure 2:**
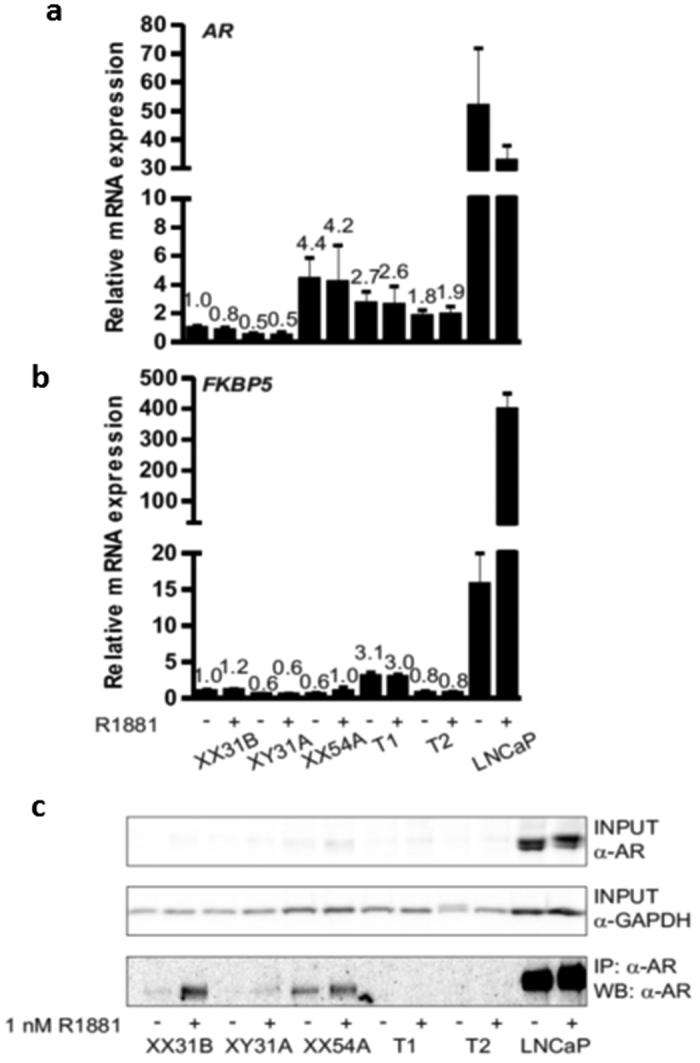
Expression of *AR* mRNA (**a**), androgen target gene *FKBP5* (**b**), and AR protein (**c**) in fibroblasts and LNCaP prostatic cancer cells. The cells were split onto 6-well plates, and after 24 h, the medium was changed to steroid-depleted medium for 6 h. A half of the wells were treated with vehicle (0.1% ethanol) (−) and a half with 1 nM R1881 (+) for 18 h before immunoprecipitation or RNA extraction. Samples XX31B, XY31A, and XX54A are control fibroblasts. The patient-derived fibroblasts are samples T1 and T2. *GAPDH* served as the reference gene for quantification of *AR* and *FKBP5* mRNA (panels a and b). The expression of all samples was normalized to the control sample XX31B vehicle treatment. The bars represent mean ± SD of 3–5 independent samples. In (**c**), AR was immunoprecipitated with rabbit polyclonal α-AR^17^ and detected in western blotting with mouse monoclonal α-AR 441 recognizing AR amino acids 299–315 in the AR N-terminal domain. α-GAPDH was used to control the loading of input samples. The samples in the different blots are from the same experiment. The blots have been cropped; full-length blots are presented in Supplementary Figure S1.

**Figure 3:**
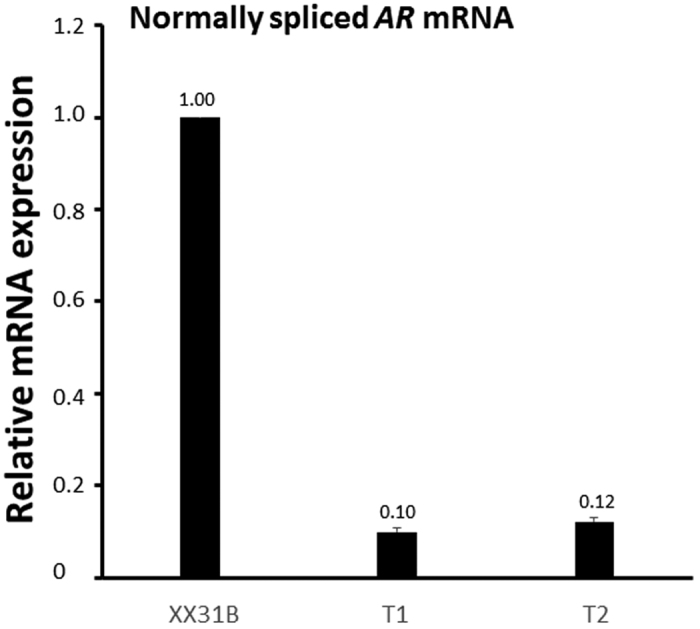
Expression of the normally spliced *AR* mRNA in fibroblasts. The normally spliced *AR* mRNA was quantified by using primers specific for the normally-spliced variant from RNA extracted from vehicle-treated (0.1% ethanol) patient-derived (T1 and T2) and control (XX31B) fibroblasts. *GAPDH* was used as the reference gene. The expression in patient samples T1 and T2 was normalized to control sample XX31B vehicle treatment. The bars represent the mean ± SD of 3 independent samples.

Both aberrant splicing events result in mRNA isoforms which code for 12 additional amino acids (NRIQLSFPLRSP) followed by a premature stop codon (PTC) after amino acid 816. Because of an intron downstream of the PTC, both aberrantly spliced mRNA isoforms are potential targets for the nonsense-mediated decay (NMD) pathway that would lead to the degradation of the AR mRNA^7^. Compared to LNCaP prostate cancer cells expressing biologically active levels of the AR, both control fibroblasts and patient samples expressed very low amounts of *AR* mRNA (≤10% of the LNCaP levels, Fig. 2a). This difference was also evident on the AR protein level (Fig. 2c). Additionally, even though the patient fibroblasts expressed overall similar amounts of the *AR* mRNA as the controls, quantification of the normally-spliced *AR* mRNA in the patient fibroblasts showed only approximately 10% level of expression as compared to the XX31B control fibroblasts (T1: 9.0–10.9% expression, T2: 11.6–12.9% expression) (Fig. 3). In accordance, immunoprecipitation with a polyclonal anti-AR antibody-coupled to western blotting with a monoclonal anti-AR antibody showed no signs of the AR protein in the patient samples as opposed to two of the control samples. The functionality of androgen signaling in the cells was also tested by examining the ability of the AR agonist R1881 to induce the expression of the AR-target gene *FKBP5*, but even in the control fibroblasts, the amount of the AR was not sufficient for any induction, whereas the induction was clearly detectable in the LNCaP cells (Fig. 2b).

## Discussion

In the current work, by combining whole-genome sequencing and cDNA analysis we were able to pinpoint the molecular genetic cause of CAIS in two 46,XY sisters with no mutations in the *AR* coding sequence or conserved splice sites. In our knowledge, although several splicing mutations of *AR* have already been reported in AIS patients, most are situated in the conserved splice sites close to the exon-intron junctions. Only one deep intronic mutation (in intron 6 located 44 bases from the exon-intron border) in *AR* has been previously reported in AIS^4^. This mutation caused diminished DHT binding capacity; however, the effect on mRNA splicing was not reported. Here, we show that the deep intronic mutation identified in this study severely disrupts the normal splicing through pseudoexon activation, a mechanism that is increasingly being recognized as a cause of human disorders^8^, but, in our knowledge, has not been earlier demonstrated in AIS. Another novel mechanism of CAIS pathogenesis was also demonstrated very recently by Hornig *et al*., who showed that a mutation in the *AR* 5′ untranslated region (UTR) created an upstream translation start site and subsequently led to the diminished expression of the full-length AR protein^9^, thus further emphasizing the importance of mutations outside the *AR* coding region in AIS.

The deep intronic mutation causes the aberrant splicing of *AR* mRNA, creating two abnormally long products and a significantly reduced amount of the normal-sized mRNA. Computational analysis and sequencing of the 800 bp product suggested that the c.2450-118A>G mutation creates a *de novo* 5′ss facilitating the binding of U1 small nuclear ribonucleoprotein (snRNP) to this sequence^10^. This in turn leads to the activation of a novel 3′ss 84 bp upstream, most likely through exon-definition interactions^11^, thus activating the intronic pseudoexon that is normally not recognized by the splicing machinery^12^. Additionally, ~50% of the crypticly spliced mRNAs show inclusion of the entire 202 nt intronic fragment downstream of the novel 3′ss (Fig. 1c,d). Computational analysis supports a model in which the patient mutation creates, in addition to the novel 5′ss, a novel exonic splicing enhancer (ESE) motif specific to the SRSF1, a member of splicing activator family (SR-proteins) that help to recruit spliceosomal components to the 3′ and 5′ splice sites^13^. As the ESE motif and 5′ss overlap, SRSF1 and U1 snRNP can compete with each other in binding to RNA, thus resulting in either a skipping or inclusion of the cryptic 5′ss, respectively, and formation of the two aberrantly spliced transcripts in approximately 1:1 ratio as observed experimentally (Fig. 1a). The upstream 3′ss is shared between the two cryptically spliced mRNA isoforms because binding of either SRSF1 or U1 snRNP can provide sufficient stimulatory interactions to activate this splice site^11^.

The inclusion of both the shorter and the longer intronic sequences is predicted to cause a premature stop codon before exon 7, which may result in degradation of the *AR* mRNA by the NMD pathway, as is predicted to occur when a premature stop codon is located at least 50–55 nt upstream of the last exon-exon boundary^7^. Unexpectedly, although quantification of the normal *AR* mRNA in the patient fibroblasts revealed only approximately 10% levels compared to the control fibroblasts, the overall abundance of *AR* mRNA was not clearly lower in the patient fibroblasts than in the controls. The destruction of the aberrant mRNA products by the NMD pathway cannot be ruled out, however, especially since the half-life of the PTC-containing mRNA products is not known; it is for example possible that some of the aberrant product is destroyed quickly, while the rest has a half-life similar to the normal *AR* mRNA^14^. Regardless, AR protein was undetectable in the patient fibroblasts, which suggests that even if the aberrantly-spliced mRNAs are translated into protein, the stability of the aberrant AR protein is compromised, and that the low amount of correct mRNA does not yield significant levels of AR protein.

In conclusion, *AR* cDNA together with intronic sequences should be examined in CAIS patients with normal coding region and conserved splice sites of *AR.* The possibility of mutations in the 5′UTR or other regulatory regions should also be taken into consideration. Other deep intronic or regulatory region mutations, often missed in routine genetic screenings, may underlie CAIS in patients who still lack a molecular genetic diagnosis. The precise molecular genetic diagnosis is of crucial importance for genetic counseling of the patients and their family members. In addition, the molecular genetic diagnosis in DSD patients is valuable in predicting the clinical course of the disease, and in decision-making related to the possible need of gonadectomy.

## Methods

### Patients

The two cases are the first (A) and the third (B) child from a non-consanguineous couple. The second child in the family is a 46,XX girl. The birth and the pregnancy of A were normal. Her birth weight was 3690 g, length 50 cm, head circumference 39.5 cm, Apgar score 9/10/10. Her growth started to follow +1.1−1.6 SDS curve. At the age of three months, she presented with an inguinal hernia on the right side. During the operation, 10 × 7 × 7 mm-size gonad was found in the hernia sac and was returned to the abdominal cavity. The vagina was short, only 0.7 cm (normally longer than 4 cm). At that time, all girls with hernias were tested for the karyotype^15^, and hers was found to be 46,XY. In the workup of the disorders of sex development (DSD), we performed ultrasound and magnetic resonance imaging (mri) and found completely feminine external genitalia, abdominal gonads without any follicular structures, and a 3–4 mm × <20 mm tubular structure behind the bladder that was considered to be a Müllerian remnant in the initial ultrasound examination, whilst the mri showed no uterine structures. Serum testosterone concentration at the age of 3 months 3 weeks was 2.5 nmol/l and inhibin-B 668 pg/ml, which verified the presence of functional testicular tissue. There were no mutations detected in the exon regions of the *AR* gene. At the age of 1.6 years, in an hCG test, Pregnyl 50 IU/kg i.m. was given on days 0, 3, and 6, and serum samples were collected before the injections and eight days after the last injection. The testosterone and the dihydrotestosterone (DHT) levels before stimulation were 1.1 nmol/l and 0.6 nmol/l, respectively, and SHBG concentration was 100 nmol/l. Serum estradiol concentration was under detection limit (0.025 nmol/l). After the stimulation, the testosterone level increased to 17 nmol/l, DHT to 1.1 nmol/l. SHBG level remained at 101 nmol/l and estradiol became measurable, 0.028 nmol/l. At the age of 10, she entered puberty with a growth spurt, breast development, and increased levels of LH (9.2 U/l), FSH (3.7 U/l), testosterone (30 nmol/l), and estradiol (0.078 nmol/l). Lanugo-type pubic hair appeared at the age of 11, and at 12 years of age she was at Tanner stage M4 P3, with scarce lanugo-type pubic hair. In annual check-ups gonads have not shown any signs of malignancy and serum hCG and alpha fetoprotein (AFP) levels have been low. Her current serum hormone levels at the age of 12.9 years are: LH 19 U/L, FSH 4.1 U/L, testosterone 29 nmol/L, estradiol 0.149 nmol/L, and inhibin B 426 ng/L, verifying functional testicular tissue.

Child B was karyotyped from the amniotic fluid and the 46,XY result was verified after the birth. Her phenotype was identical to A’s. No uterine structures were found in mri. The pregnancy and the birth were normal, size at birth 3860 g, 53 cm, head circumference 35.5 cm, and Apgar score 9/10/10. At 5 days age, the serum testosterone level was 1.2 nmol/l, DHT under 0.2 nmol/l, LH less than 0.1 U/l, FSH 0.24 U/l. At 2.5 months, testosterone level was surprisingly less than 0.5 nmol/l, but DHT level was 0.2 nmol/l; LH 0.15 U/l and FSH 0.76 U/l. The low testosterone level is consistent with a previous report of absent postnatal T surge in CAIS patients^16^. Her growth has followed +1SDS curve. She has not yet reached puberty at the age of eight years. There have been no signs of gonadal malignancies in annual ultrasound examinations of the gonads, and her hCG and AFP levels have remained low. Her current serum hormone levels at the age of 8.9 years are LH 3.2 U/L, FSH 4.4 U/L, testosterone 1.1 nmol/L, estradiol <0.025 nmol/L, and inhibin B 222 ng/L. These values are consistent with the presence of functional testicular tissue and commencing puberty.

The parents and the children have given their informed consent and assent, respectively, to the study that was approved by the Ethics committee of Helsinki University Central Hospital. The study was carried out in accordance with the approved guidelines.

### Sequencing

Genomic DNA was extracted from the peripheral blood leukocytes of the subjects. The entire coding region (exons 1–8) and the exon-intron junctions of *AR* (RefSeq ID NM_000044.3) were PCR-amplified from the genomic DNA of Child A and Child B and the PCR products were purified with ExoSAP-IT treatment (Amersham Biosciences, Piscataway, NJ), and bi-directionally sequenced by using the ABI BigDyeTerminator Cycle Sequencing Kit (v3.0) and ABI 3730xl 96-capillary DNA Analyzer automated sequencer (Applied Biosystems, Foster City, CA). The sequences were aligned and read with Sequencher 4.9 software (Gene Codes Corporation, Ann Arbor, MI). All primer sequences and PCR conditions are available upon request.

The whole-genome sequencing of the two patients and their father, who served as a healthy control, was performed in the Beijing Genomic Institute (BGI, Shenzhen, China) by using Illumina’s HiSeq 2000 technology. The intronic *AR* variant found in the genome-sequencing data was confirmed by Sanger-sequencing from all family members.

### Cell culture

Human control and patient fibroblasts (obtained from the labia majora) were grown in DMEM (Gibco, Life Technologies, 41965, Paisley, Scotland) supplemented with 1% (vol/vol) penicillin and streptomycin and 10% fetal calf serum (FCS; HyClone, Thermo Scientific SV30160-03, Gramlington, UK). LNCaP prostatic cancer cells were used as a control for AR expression and androgen response and were grown in RPMI (Gibco, Life Technologies, A10491, Grand Island, NY, USA) supplemented with 1% (vol/vol) penicillin and streptomycin and 10% FCS. For immunoprecipitations and RNA extractions, the cells were split onto 6-well plates (250 000 cells/well) and allowed to grow for 24 h. Medium was changed to steroid-depleted medium (for fibroblasts: DMEM supplemented with 2.5% stripped serum and for LNCaP: RPMI supplemented with 10% stripped serum) for 6 h and a half of the wells were treated with vehicle (0.1% EtOH) and a half with 1 nM AR agonist methyltrienolone (R1881) (Perkin Elmer, NCP-005, Boston, MA, USA) for the next 18 h.

### Immunoprecipitation and western blotting

Immunoprecipitation was performed with Magna ChIP™ Protein A magnetic beads (Millipore, Temecula, CA, USA) from protein extract of 2 million cells. Beads needed for preclearing were equilibrated in lysis buffer (50 mM Tris-HCl, pH 8.0, 140 mM NaCl, 1 mM EDTA, 1% (vol/vol) Triton X-100, 10% glycerol, 10 mM Na-phosphate, 50 mM NaF, 10 mM N-ethylmaleimide (NEM), 1x protease inhibitor cocktail (PIC) (cOmplete Protease Inhibitor Cocktail tablets, Roche Diagnostics GmbH, Mannheim, Germany)) which contained 0.5% bovine serum albumin (BSA). Immunoprecipitating polyclonal antibody (rabbit α-AR)^17^ raised against full-length AR was coupled to beads in lysis buffer which contained 0.5% BSA in rotation over night at +4 °C.

Cells were harvested (three wells in one sample) in PBS containing 10 mM NEM and suspended in lysis buffer. After 20 min incubation on ice, the samples were sonicated 10 sec and centrifuged 13000 × g for 20 min at +4 °C. Supernatants were transferred into clean tubes and precleared with equilibrated magnetic beads in rotation at +4 °C for 1 h. The beads and the supernatant were separated with a magnet, and an aliquot was taken into a separate tube as an input. The rest of the supernatants were immunoprecipitated with antibody-coupled beads in rotation at +4 °C overnight. The magnetic beads were washed three times with lysis buffer. Immunoprecipitated proteins were eluted from the beads by 1 × SDS sample buffer containing 10 mM NEM and 1 × PIC. The inputs and the immunoprecipitated samples were run on PAGE gels and transferred onto nitrocellulose membranes. The amount loaded into each lane corresponded to 40% of each immunoprecipitate. AR was recognized by mouse monoclonal α-AR antibody 441 (targeted against AR N-terminal domain amino acids 299–315, sc-7305, Santa Cruz Biotechnology Inc., Santa Cruz, CA, USA) and HRP-conjucated anti-mouse secondary antibody (Life Technologies, Eugene, OR, USA). Membranes were detected also by rabbit polyclonal α-GAPDH antibody (sc-25778; Santa Cruz Biotechnology Inc.) to determine the loading of the input samples.

### RNA extraction and RT-qPCR analysis

Total RNA was extracted by using TriPure Isolation Reagent (Roche) according to the manufacturer’s instructions. RNA pellets were suspended in 30 μl of sterile H_2_O and 1 μg was used for cDNA synthesis in 20 μl volume by using Transcriptor First Strand cDNA Synthesis Kit (Roche Diagnostics). cDNA samples were diluted with sterile H_2_O into 200 μl. An aliquot (4 μl) was used in a qPCR reaction containing 0.4 μM each primer and 1 × LightCycler® 480 SYBR Green I Master (Roche) in 10 μl reaction volume by using the following PCR procedure: denaturation at 95 °C for 10 min, 40 cycles of 95 °C, 20 sec; 58 °C, 20 sec and 72 °C, 20 sec, and final extension 72 °C for 5 min. Primer sequences were for *AR*: forward 5′-TTGGAGACTGCCAGGGAC-3′ and reverse 5′-TCAGGGGCGAAGTAGAGC-3′, for *FKBP5*: forward 5′-AAAAGGCCAAGGAGCACAAC-3′ and reverse 5′-TTGAGGAGGGGCCGAGTTC-3′, for *GAPDH*: forward 5′-TGGGGAAGGTGAAGGTCGG-3′ and reverse 5′-TCTCAGCCTTGACGGTGCC-3′. Quantification of the normally-spliced *AR* mRNA was performed with primers targeting specifically the normal variant: forward 5′-CAGTGTGTCCGAATGAGGCA-3′ and reverse (located at the junction of exons 6 and 7) 5′-CCCATCCACTGGAATAATGCTGA-3′. The results were calculated for each gene of interest in relation to *GAPDH* expression. Further, the results of all samples were expressed in relation to control sample XX31B vehicle (0.1% ethanol) treatment (value set to 1) using the formula 2^−(ΔΔCt)^, where ΔΔC_t_ is ΔC_t(sample)_−ΔC_t(XX31B vehicle)_, ΔC_t_ is C_t(gene of interest)_−C_t(GAPDH)_ and C_t_ is the cycle where the threshold value is crossed.

### Sequencing of AR cDNA

The entire coding region of *AR* was PCR-amplified from the cDNA with cDNA-specific primers in overlapping fragments. The PCR products were visualized on a 1.5% agarose gel. In lanes with multiple-sized PCR products, the products were extracted from the gel with QIAquick gel extraction kit (Qiagen, Hilden, Germany) for sequencing. All PCR products were sequenced as described above.

### *In silico* prediction

The effects of the identified intronic mutation were predicted with the web-based analysis tool Human Splicing Finder v.3.0^18^. The analysis was performed with the full sequence of intron 6 and the flanking exons using the default settings.

## Additional Information

**How to cite this article**: Känsäkoski, J. *et al*. Complete androgen insensitivity syndrome caused by a deep intronic pseudoexon-activating mutation in the androgen receptor gene. *Sci. Rep.* **6**, 32819; doi: 10.1038/srep32819 (2016).

## Supplementary Material

Supplementary Information
