# Supplementary material for: Complete androgen insensitivity syndrome caused by a deep intronic pseudoexon-activating mutation in the androgen receptor gene
Source: Sci Rep. 2016 Sep 9;6:32819. doi: 10.1038/srep32819 (PMC5016895; doi:10.1038/srep32819)
Supplement: Supplementary Information [file srep32819-s1.pdf]

Supplementary information

Complete androgen insensitivity syndrome caused by a deep intronic pseudoexon-activating mutation in the androgen receptor gene

Johanna Känsäkoski[1,2], Jarmo Jääskeläinen[3], Tiina Jääskeläinen[4], Johanna Tommiska[1,2], Lilli Saarinen[5], Rainer Lehtonen[5], Sampsa Hautaniemi[5], Mikko J. Frilander[6], Jorma J. Palvimo[7], Jorma Toppari[8†], Taneli Raivio[1,2†]

[1] Physiology, Faculty of Medicine, University of Helsinki, Helsinki, Finland

[2] Children's Hospital, Helsinki University Hospital, Helsinki, Finland

[3] Department of Pediatrics, University of Eastern Finland and Kuopio University Hospital, Kuopio, Finland

[4] Institute of Dentistry and Institute of Biomedicine, University of Eastern Finland, Kuopio, Finland.

[5] Research Programs Unit, Genome-Scale Biology, Faculty of Medicine, University of Helsinki, Helsinki, Finland

[6] Institute of Biotechnology, University of Helsinki, Helsinki, Finland

[7] Institute of Biomedicine, University of Eastern Finland, Kuopio, Finland

[8] Departments of Physiology and Pediatrics, University of Turku and Turku University Hospital, Finland

[†]Equal contribution

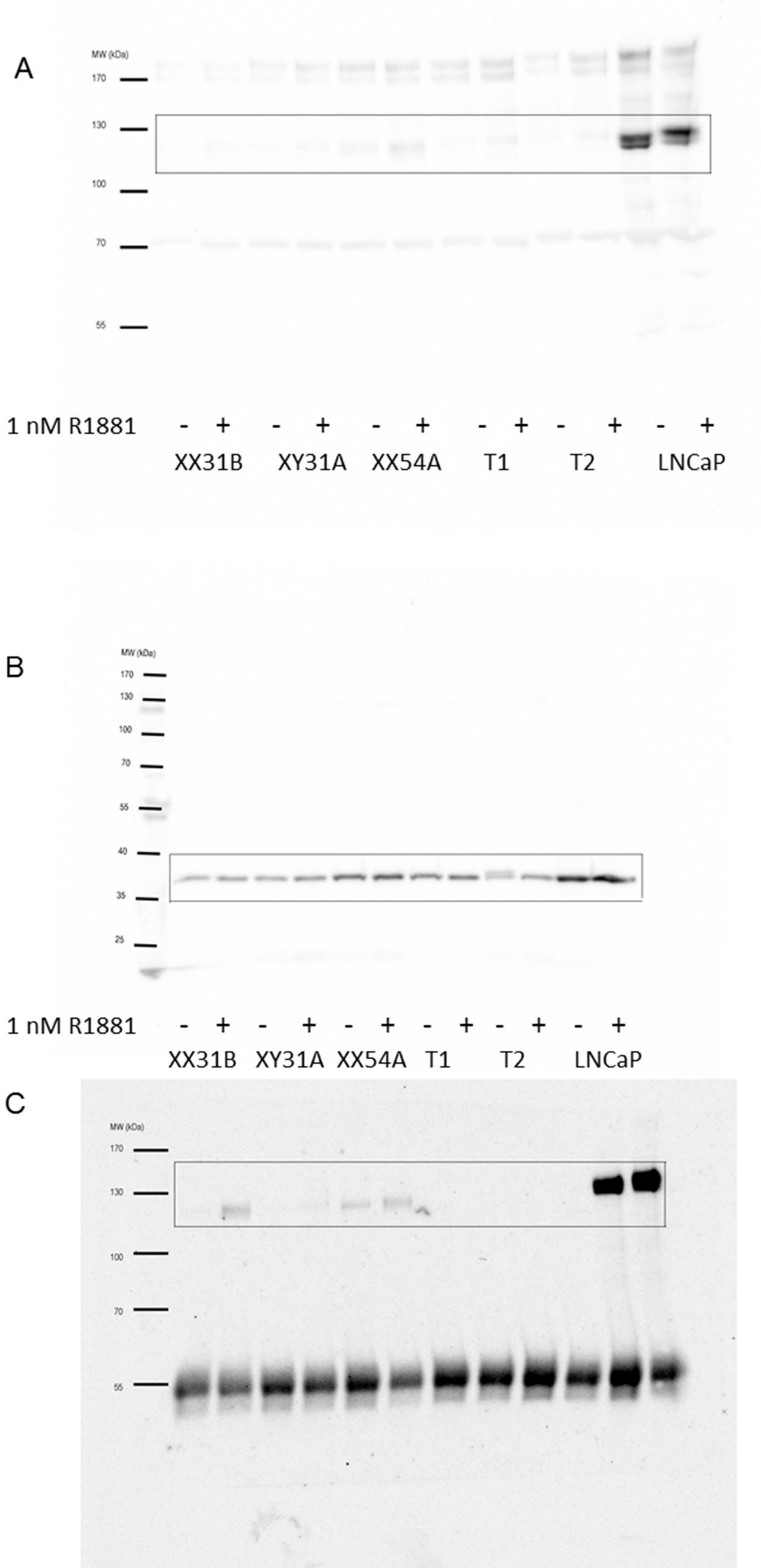

Supplementary Figure S1. Full-length blots of AR protein expression analysis. Areas of the blots that are presented in Fig. 2 are marked with a box. A) Input samples for AR immunoprecipitation detected with mouse

monoclonal α-AR antibody. B) Input samples for AR immunoprecipitation detected with α-GAPDH antibody to control the loading of input samples. C) Immunoprecipitated samples detected with mouse monoclonal α-AR antibody.
